# Agonist activation of estrogen receptor beta (ERβ) sensitizes malignant pleural mesothelioma cells to cisplatin cytotoxicity

**DOI:** 10.1186/1476-4598-13-227

**Published:** 2014-10-02

**Authors:** Giulia Pinton, Arcangela G Manente, Antonio Daga, Michele Cilli, Maurizio Rinaldi, Stefan Nilsson, Laura Moro

**Affiliations:** Department of Pharmaceutical Sciences, University of Piemonte Orientale “A. Avogadro”, Lgo Donegani 2, 28100 Novara, Italy; IRCCS San Martino-IST, 16132 Genova, Italy; Karo Bio AB, Novum, S-141 57 Huddinge, Sweden; Department of Biosciences and Nutrition, Karolinska Institutet, Novum, S-141 57 Huddinge, Sweden

**Keywords:** Estrogen Receptor β, Malignant mesothelioma, Receptor agonist, Cisplatin, Therapy

## Abstract

**Background:**

Estrogen receptor (ER) β acts as a tumor suppressor in malignant mesotheliomas.

**Methods:**

Here we explored the anti-proliferative and anti-tumorigenic efficacy of the selective ERβ agonist, KB9520, in human mesothelioma cell lines *in vitro* and in a mesothelioma mouse model *in vivo*.

**Results:**

KB9520 showed significant anti-proliferative effect in ERβ positive human malignant pleural mesothelioma cells *in vitro*. Selective activation of ERβ with KB9520 sensitized the cells to treatment with cisplatin, resulting in enhanced growth inhibition and increased apoptosis. Furthermore, in CD1 nude mice mesothelioma tumor growth was significantly inhibited when KB9520 was added on top of the standard of care chemo combination cisplatin/pemetrexed, as compared to the cisplatin/pemetrexed alone group. Importantly, KB9520 exerted a protective effect to cisplatin toxicity in the non-malignant mesothelium derived MET5A cells.

**Conclusions:**

Together, the data presented suggest that selective targeting of ERβ may be an efficacious stand-alone treatment option and/or become an important add-on to existing malignant mesothelioma therapy.

**Electronic supplementary material:**

The online version of this article (doi:10.1186/1476-4598-13-227) contains supplementary material, which is available to authorized users.

## Background

Malignant pleural mesothelioma (MPM) is an aggressive cancer associated with exposure to asbestos. Currently rates of MPM are rising and estimates indicate that the incidence of MPM will peak within the next 10–15 years in the western world, while in Japan the peak is predicted not to occur until 40 years from now [1, 2]. Although the use of asbestos has been banned in many countries around the world, production of and the potentially hazardous exposure to asbestos is still present with locally high incidences of mesothelioma [3]. Today a new man-made material, carbon nanotubes (CNTs), has arisen as a concern; CNTs may display ‘asbestos-like’ pathogenicity with mesothelioma induction potential [4, 5]. The pharmacology of CNTs, including effects on immune responses and tissue accumulation, is therefore intensely investigated; “it is better to be safe than sorry” [6].

MPM is an extremely difficult disease to treat, with a median overall survival ranging between 9 and 17 months, regardless of disease stage [7, 8]. The combination of pemetrexed and cisplatin has been established as the current standard of care (SOC) but only 40% of treated patients show response to this therapy, with an overall median survival of 12.1 months [9].

There is an urgent need for new targeted therapy for clinical management of MPM that can stop progression and stabilize the disease or ideally erase the tumor, either as add-on to SOC, to improve treatment efficacy and reduce chemoresistance and toxicity, or as monotherapy for patients whose performance status does not allow aggressive treatment.

Rapid advances in the understanding of cancer biology are leading to identification of new targets for cancer treatment and personalized therapy is rapidly becoming a reality with the aim of improving survival and quality of life for cancer patients [10–12].

The expression of estrogen receptor beta (ERβ) in malignant pleural and peritoneal mesothelioma correlates with longer patient survival and is an independent prognostic factor [13, 14]. Our group recently demonstrated that 70-80% of MPMs express ERβ, which acts as a tumor suppressor, inhibiting MPM cell proliferation and invasiveness [13, 15].

ERβ is the second ER subtype identified in several human tissues traditionally believed to be ER negative [16]. ERβ is expressed and demonstrated to exert anti-proliferative effects in different preclinical *in vitro* and *in vivo* models of human cancers, for example, breast, colon, prostate, lymphoma, and pleural mesothelioma [13, 15, 17–25]. Moreover, ERβ is proposed to mediate the beneficial clinical effects in ERα negative breast cancer patients [26], in malignant intraperitoneal mesothelioma [27], and in the prevention of colon cancer in women on menopausal hormone therapy (MHT) [28].

Drugs that selectively target ERβ might be safer than non-selective estrogens, which are associated with increased risk of breast, endometrial and ovarian cancer in women and the development of prostate cancer in men. These serious side effects of non-selective estrogens are mediated by the ERα subtype [29–32]. Several synthetic and natural ERβ-selective compounds have been identified [19, 33–36] that have shown promising anti-tumorigenic efficacy in preclinical cancer models [37–43]. Therefore, drugs with selectivity for ERβ might prove promising in the the development of novel, targeted therapies for the clinical management of human cancers.

In the present study, we characterized the efficacy of KB9520, a selective ERβ agonist, to inhibit MPM cell growth *in vitro* and *in vivo*. Moreover, we investigated the possibility of an additive or synergistic effect between KB9520 and the SOC regimen (cisplatin/pemetrexed) for treatment of MPM.

## Results

### KB9520-mediated ERβ activation affects MPM cell proliferation

**Figure 1:**
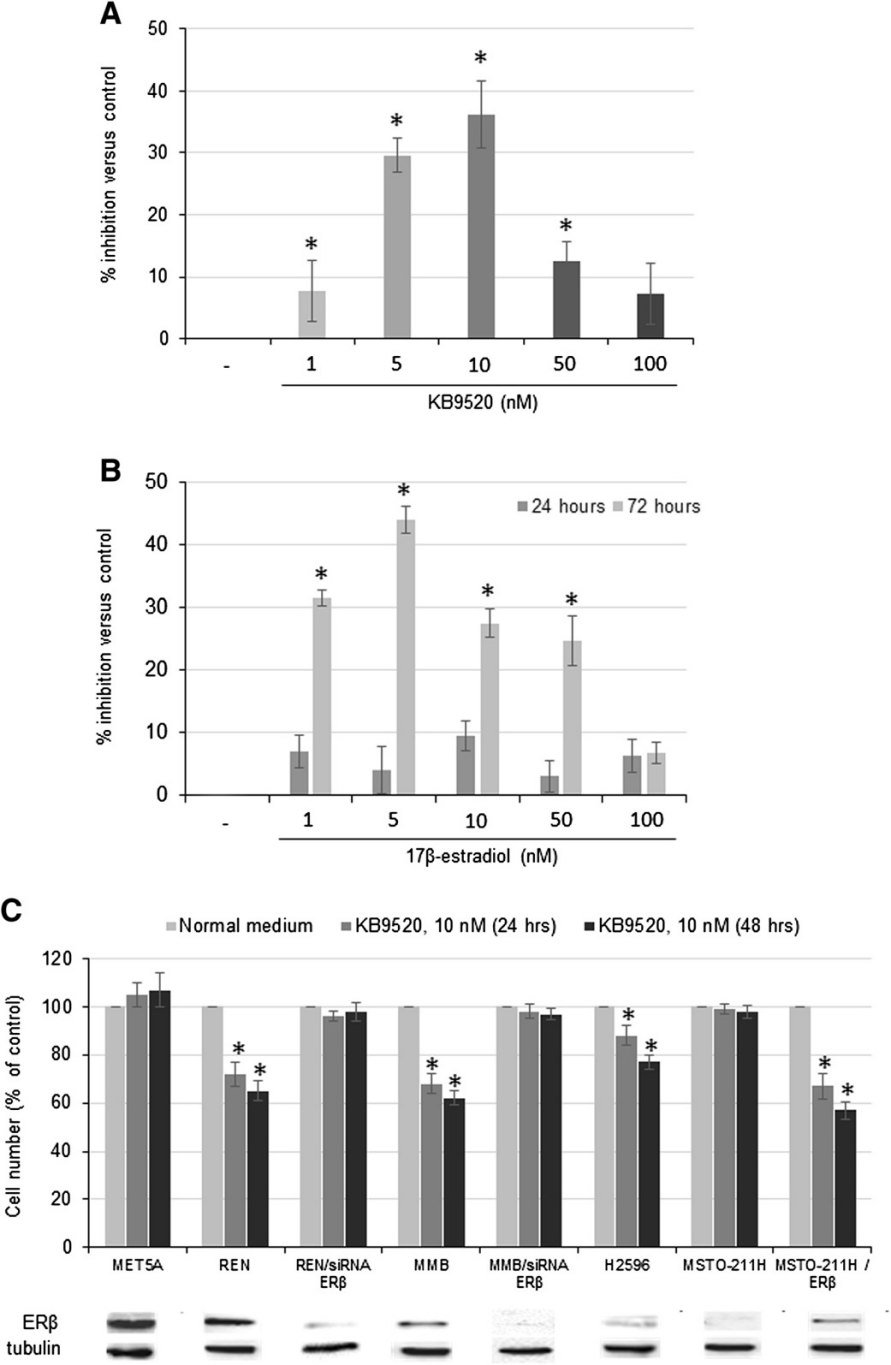
**KB9520 mediated ERβ activation affects MPM cells proliferation. A)** Bar graph shows the percentage of growth inhibition of REN cells after 24 hours treatment with different concentrations of KB9520 (range 1–100 nM) versus untreated cells. **B)** Bar graph shows the percentage of growth inhibition of REN cells after 24 and 72 hours treatment with different concentrations of 17β-estradiol (range 1–100 nM) versus untreated cells. **C)** Bar graph shows the percentage of growth inhibitory effect of 10 nM KB9520 at 24 and 48 hours in mesothelium derived cells (MET5A), mesothelioma cells with different levels of endogenous ERβ expression (REN, MMB, H2596 and MSTO-211H), ERβ silenced REN and MMB cells (REN/siRNA ERβ, MMB/siRNA ERβ) and MSTO-211H/ERβ cells, which were transiently transfected to express human ERβ. Western blots, below the bar graph, show ERβ protein expression for each cell line. Tubulin was included as a loading control. Each graph is representative of three independent experiments. Each bar represents mean ± s.d. *p ≤ 0.05.

The growth inhibitory effect of different concentrations of KB9520 (range 1–100 nM) was tested on ERβ positive REN mesothelioma cells (Figure 1A). KB9520 significantly (p ≤ 0,05) reduced cell growth and viability in a concentration-dependent manner, with highest efficacy at 10 nM. For comparison we also examined the concentration-dependent growth inhibitory effect of 17β-estradiol (E2) on REN cells (Figure 1B). The anti-proliferative activity of KB9520 as single agent was then assessed in the non-malignant mesothelium derived MET5A cells and in the malignant mesothelioma REN, MMB, H2596, and MSTO-211H cell lines, respectively (Figure 1B). KB9520 significantly (p ≤ 0,05) inhibited proliferation of the REN and MMB cells, whereas no inhibitory effect was observed in the MET5A cells, despite high endogenous levels of ERβ. The H2596 cells express very low levels of ERβ which explains their weaker response to KB9520. As expected, the ERβ negative MSTO-211H cells and the ERβ silenced REN and MMB cells showed no response to KB9520 treatment. However, transient transfection of the MSTO-211H cells with an ERβ expression vector sensitized also these cells to KB9520 (Figure 1B). In addition, increasing the expression of ERβ in H2596 by transient transfection with an ERβ expression vector increased the response to KB9520 also in these cells (data not shown).

### KB9520 significantly increases the effect of cisplatin/pemetrexed *in vitro*and *in vivo*

**Figure 2:**
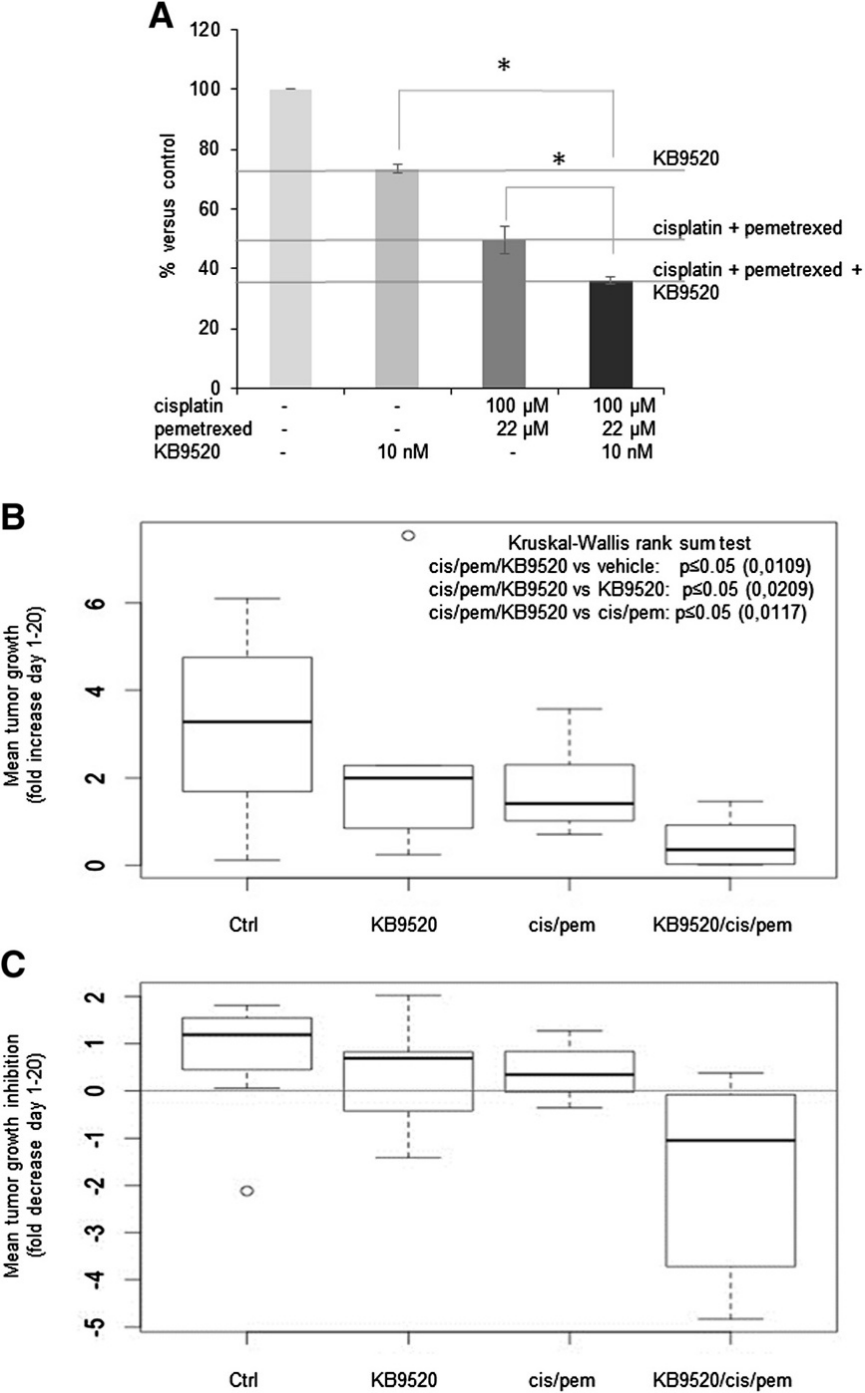
**KB9520 significantly increases cisplatin/pemetrexed effects** ***in vitro*** **and** ***in vivo*** **. A)** Percentage of viable REN cells exposed for 24 hours to 10 nM KB9520 alone or in combination with cisplatin (100 μM) and pemetrexed (22 μM), versus untreated cells. Graph is representative of three independent experiments. Each bar represents mean ± s.d. *p ≤ 0.05. **B)** and **C)** Box plots of the 4 different treatment groups (10 mice/group) showing *in vivo* mean tumor growth **(B)** and mean tumor growth inhibition **(C)** evaluated after 21 days of treatment. The thick segments represent the medians while the upper and lower borders of each rectangle represent the quartiles. Bars show minimum and maximum values for each group, outliers are identified by a small circle. Kruskal-Wallis rank sum test (in **B**) confirms that combination treatment significantly reduced tumor growth, compared with control or single treatments.

To test if KB9520 influences MPM cell response to chemotherapy *in vitro*, we explored the effect of adding 10 nM KB9520 to the cisplatin/pemetrexed chemo combination (at their respective IC50 concentrations) on REN cell viability. As shown in Figure 2A, the triple combination KB9520/cisplatin/pemetrexed (10 nM/100 μM/22 μM, respectively) was superior (p ≤ 0.05) to either KB9520 or cisplatin/pemetrexed treatment alone.

To confirm the role of agonist activated ERβ in the enhanced response to chemotherapy, the effect of KB9520 on top of the cisplatin/pemetrexed combination was evaluated also in the mesothelioma *in vivo* mouse model. Six weeks old CD1 nude male mice were inoculated intra peritoneum with 2×10^6^ REN cells (4 groups, 10 animals per group). Prior to inoculation, the MPM cells were transduced with a lentiviral vector carrying the luciferase gene, to allow imaging in live mice. Treatment of the animals was initiated fifteen days after cell inoculation when tumor take-rate in the peritoneal cavity was 100% in all animal groups. The ERβ-selective agonist KB9520 was administrated on day 15 through 35 by subcutaneous injection at 10 mg/kg/day. Untreated animals were subcutaneously dosed with empty vehicle. Two groups were treated at day 18 and 25 with 5 mg/kg cisplatin followed by 5 days treatment with 150 mg/kg pemetrexed (days 19–23 and 26–30), alone or in combination with KB9520 (see the treatment schedule in Table 1). KB9520 alone treated mice produced a similar decrease in tumor dimensions as the cisplatin/pemetrexed treated group compared to vehicle controls at day 10 (data not shown). After 21 days of treatment, we observed a statistically significant reduction in tumor growth in the group treated with KB9520 plus cisplatin/pemetrexed as compared to the vehicle, KB9520, and cisplatin/pemetrexed groups, respectively (Figure 2B, C). Furthermore, these statistical significant differences were confirmed when the tumor growth curves were compared. Treatment with KB9520 was not toxic as assessed by monitoring changes of mice body weights during drug administration (data not shown). 35 days after MPM cell inoculation all animals were sacrificed and tumors were dissected and immediately frozen.

**Table 1:** **Treatment schedule of the** ***in vivo*** **experiment**

Day 1	Treatment	Day 15	16	17	18	19	20	21	22	23	24	25	26	27	28	29	30	31	32	33	34	Day 35
REN cells injected	KB9520 (10 mg/kg)	X	X	X	X	X	X	X	X	X	X	X	X	X	X	X	X	X	X	X	X	X
	cisplatin (5 mg/kg)				X							X										
	pemetrexed (150 mg/kg)					X	X	X	X	X			X	X	X	X	X					

### Biological sustainability of KB9520

**Figure 3:**
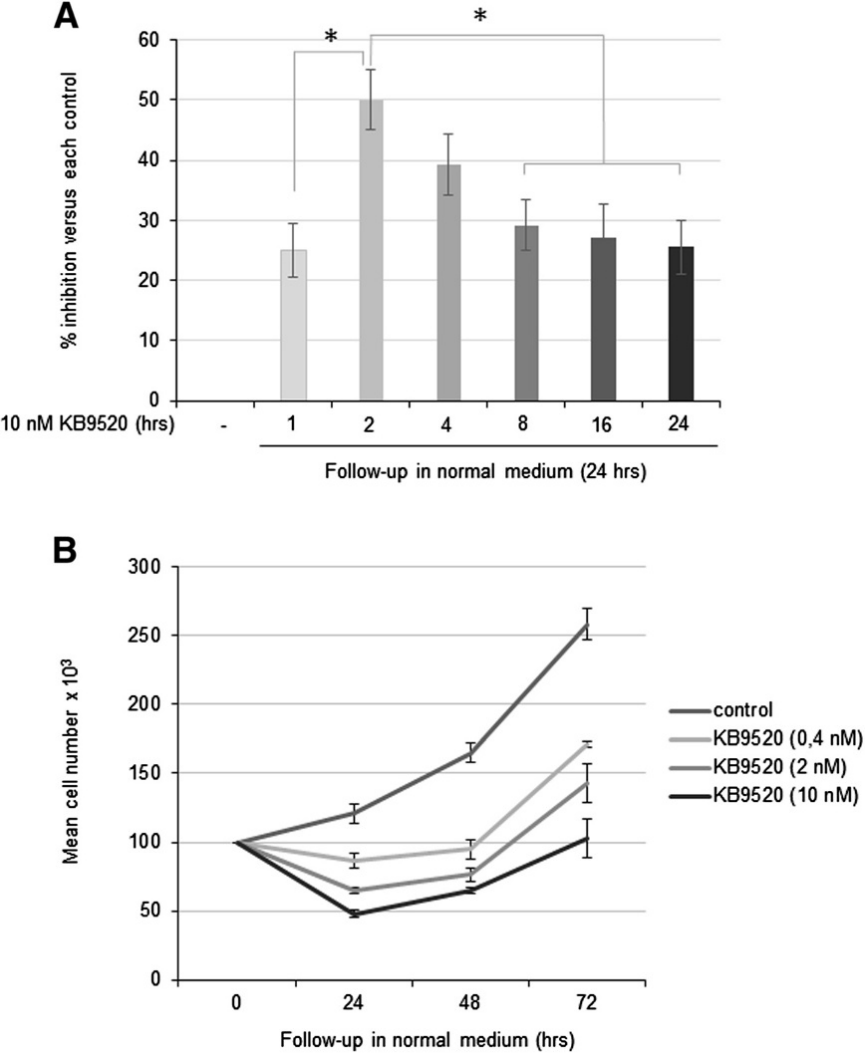
**Biological sustainability of KB9520. A)** Percentage of growth inhibition in REN cells after 1, 2, 4, 8, 16 or 24 hours pre-treatment with 10 nM KB9520 followed by wash-off and continued growth in normal medium for an additional 24 hours. **B)** Mean number of REN cells pre-exposed for 2 hours to normal medium or 0.4, 2, 10 nM KB9520 followed by wash-off and continued growth for additional 24-, 48- and 72 hours in normal medium, respectively. Each graph is representative of three independent experiments. Each point represents mean ± s.d. *p ≤ 0.05.

Since the *in vivo* plasma half-life of KB9520 is only approximately 1 hour in mice (data not shown) we decided to investigate the biological sustainability and mechanism of action of KB9520 *in vitro* to better understand its anti-tumorigenic activity and synergism with cisplatin/pemetrexed *in vivo*. Firstly, we tested the anti-proliferative response to brief exposures (1, 2, 4, 8, 16 and 24 hours) to 10 nM KB9520 on the ERβ positive REN cells (Figure 3A). An exposure of 2 hours presented significantly (p ≤ 0.05) increased inhibitory activity relative to 1 hour exposure and exposures longer than 8 hours. Successively, the 2 hours exposure to KB9520 was characterized further. Cells were treated with different concentrations of KB9520 (0.4, 2 and 10 nM) for 2 hours followed by wash-off and continued growth in normal medium (without KB9520) for an additional 24, 48 and 72 hours (Figure 3B). Control cultures were maintained in normal medium only. The duration of inhibitory effect on REN cell proliferation sustained for at least 24 hours irrespective of concentration of KB9520 used in the 2-hours pre-treatment period. The largest anti-proliferative effect was, however, observed with the highest KB9520 concentration used. After 24 hours the cells slowly regained proliferative activity and from ~48 hours post KB9520 pre-treatment, their proliferative rates were similar to that of REN cells cultivated in normal medium from start of study.

### KB9520 pre-treatment sensitizes REN cells to cisplatin

**Figure 4:**
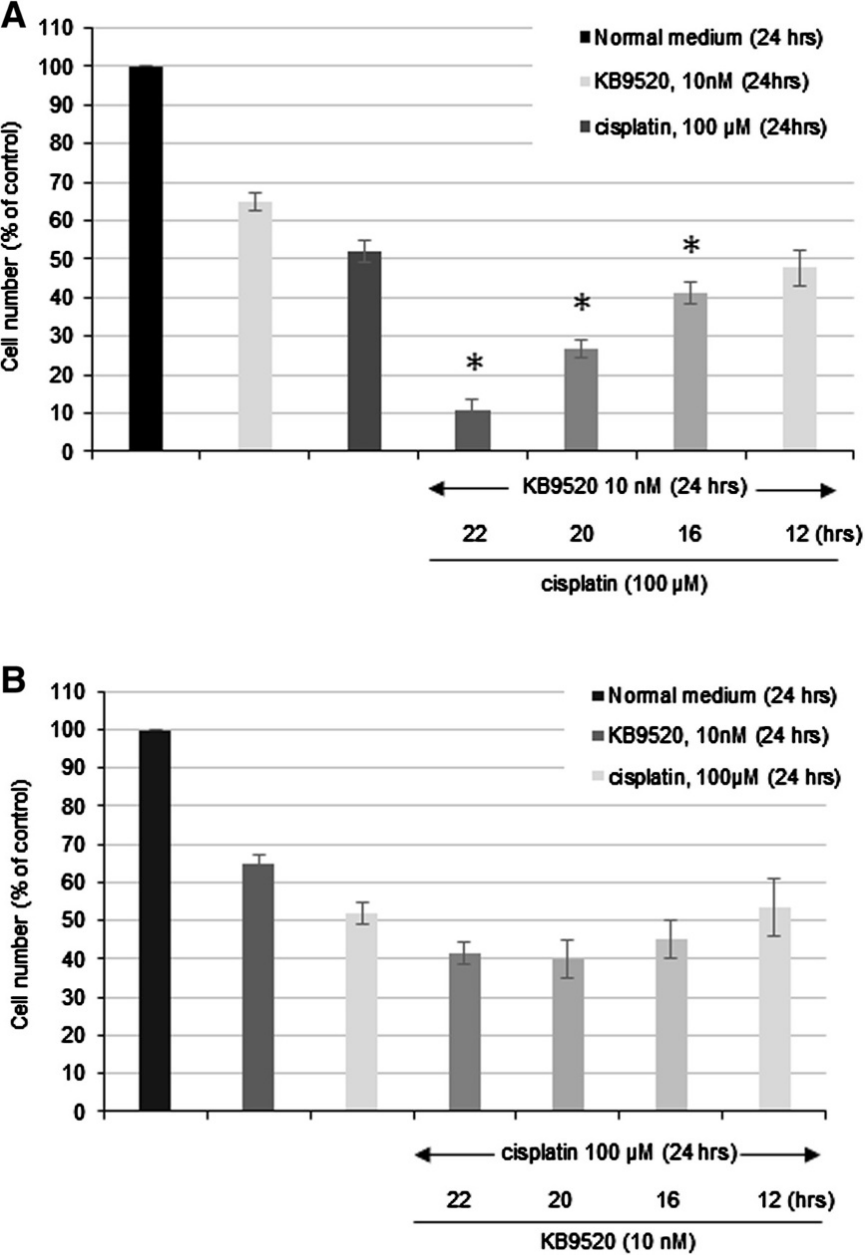
**KB9520 pre-treatment synergizes with cisplatin. A)** Effect of adding of cisplatin (100 μM) 2, 4, 8 or 12 hours after start of KB9520 (10 nM) treatment on REN cell viability. **B)** Effect of adding KB9520 (10 nM) 2, 4, 8 or 12 hours after start of cisplatin treatment (100 μM) on REN cell viability. Each graph is representative of three independent experiments. Each point represents mean ± s.d. *p ≤ 0.05.

**Figure 5:**
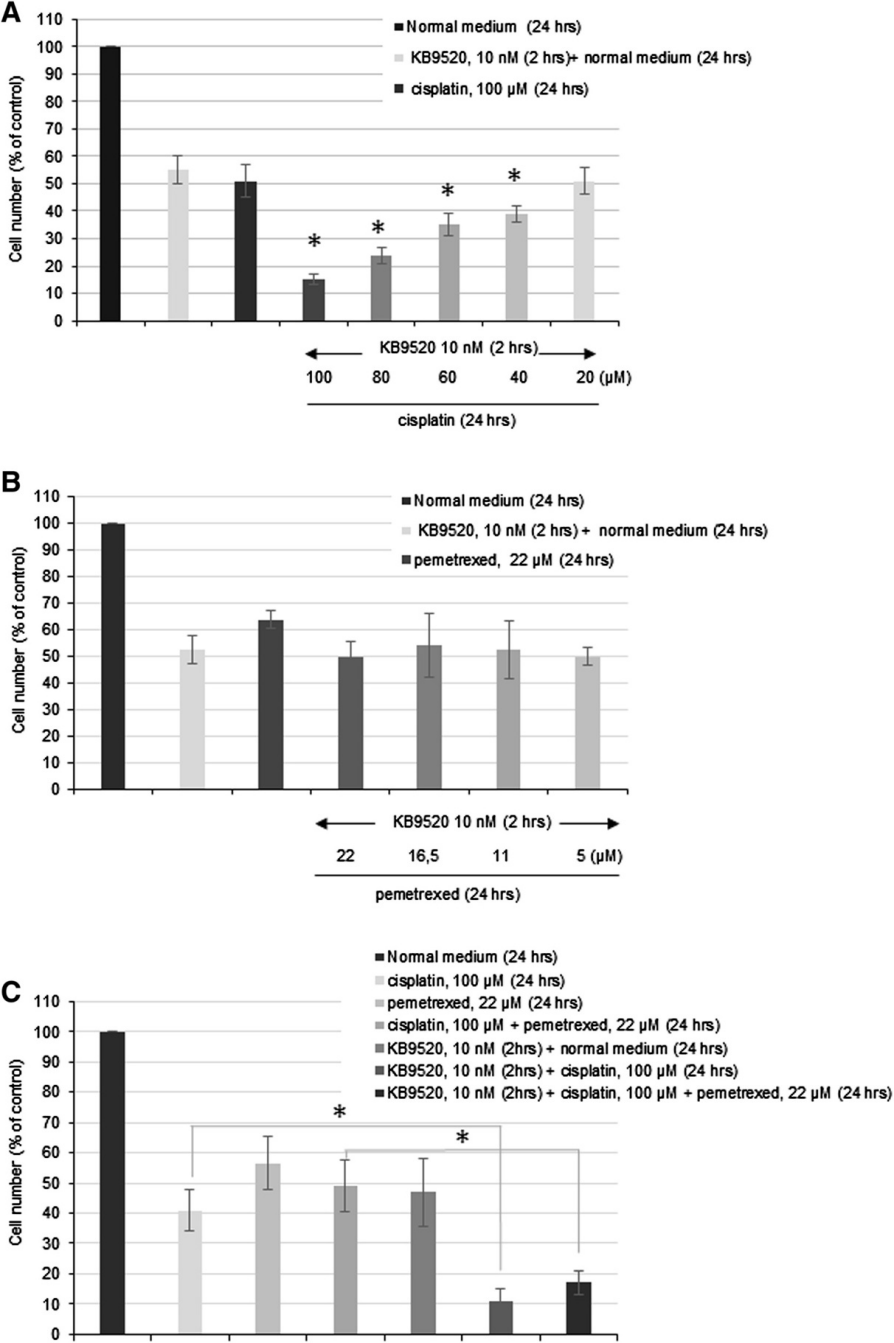
**KB9520 sensitizes REN cells to cisplatin and the standard of care chemo combination.** Effect on REN cell viability after 2 hours pre-treatment with KB9520 (10nM) followed by wash-off and continued growth in normal medium supplemented with different concentrations of **A)** cisplatin (20–100 μM), for additional 24 hours, **B)** pemetrexed (5–22 μM), for additional 24 hours and **C)** cisplatin (100 μM) or the cisplatin (100 μM)/pemetrexed (22 μM) combination, for an additional 24 hours. Each graph is representative of three independent experiments. Each point represents mean ± s.d. *p ≤ 0.05.

To explore the sequence of drug administration and its effect on cell proliferation we performed add-on or wash-off experiments (Figures 4 and 5). In the first study 100 μM cisplatin was added to REN cell cultures pre-treated for 2, 4, 8 and 12 hours with 10 nM KB9520 (Figure 4A). The enhanced anti-proliferative effect of cisplatin was time-dependent with the greatest inhibitory effect obtained adding cisplatin within 2 hours of KB9520 pre-treatment.

The reverse order of pre-treatment starting with cisplatin for 2, 4, 8 and 12 hours, respectively, prior to adding 10 nM KB9520, was also investigated (Figure 4B). In contrast to pre-treatment with KB9520 before adding cisplatin, pre-treatment with cisplatin prior to KB9520 addition did not result in synergistic inhibition of cell growth and viability.

Next we explored the combination of 2 hours KB9520 pre-treatment with different concentrations of cisplatin (range 20–100 μM) (Figure 5A). The most efficacious anti-proliferative effect was observed when KB9520 pre-treatment was combined with the highest concentration of cisplatin (100 μM). Interestingly, 2 hours pre-treatment with KB9520 in combination with 20 μM cisplatin was as efficacious as 100 μM cisplatin alone.

Also the effect of combining KB9520 with pemetrexed was explored *in vitro* (Figure 5B). Results of 2 hours pre-treatment with 10 nM KB9520, wash-off and then continued growth in normal medium, or medium supplemented with pemetrexed at different concentrations (range 5–22 μM) for an additional 24 hours are shown. No synergism or additive effect was observed with KB9520 in combination with pemetrexed compared to KB9520 or pemetrexed alone. Finally, the effect of pre-treating REN cells with 10 nM KB9520 for 2 hours prior to adding the cisplatin/pemetrexed chemo combination was explored *in vitro* (Figure 5C). As shown, adding cisplatin or the cisplatin/pemetrexed combination to REN cells pre-treated for 2 hours with KB9520 resulted in a strong synergistic inhibitory effect on REN cell growth and viability.

In summary, our data suggest that KB9520 acts as a chemosensitizer through ERβ, increasing the anti-tumorigenic efficacy of cisplatin or the cisplatin/pemetrexed combination on malignant mesothelioma.

### Mechanism of KB9520 sensitization to cisplatin cytotoxicity

**Figure 6:**
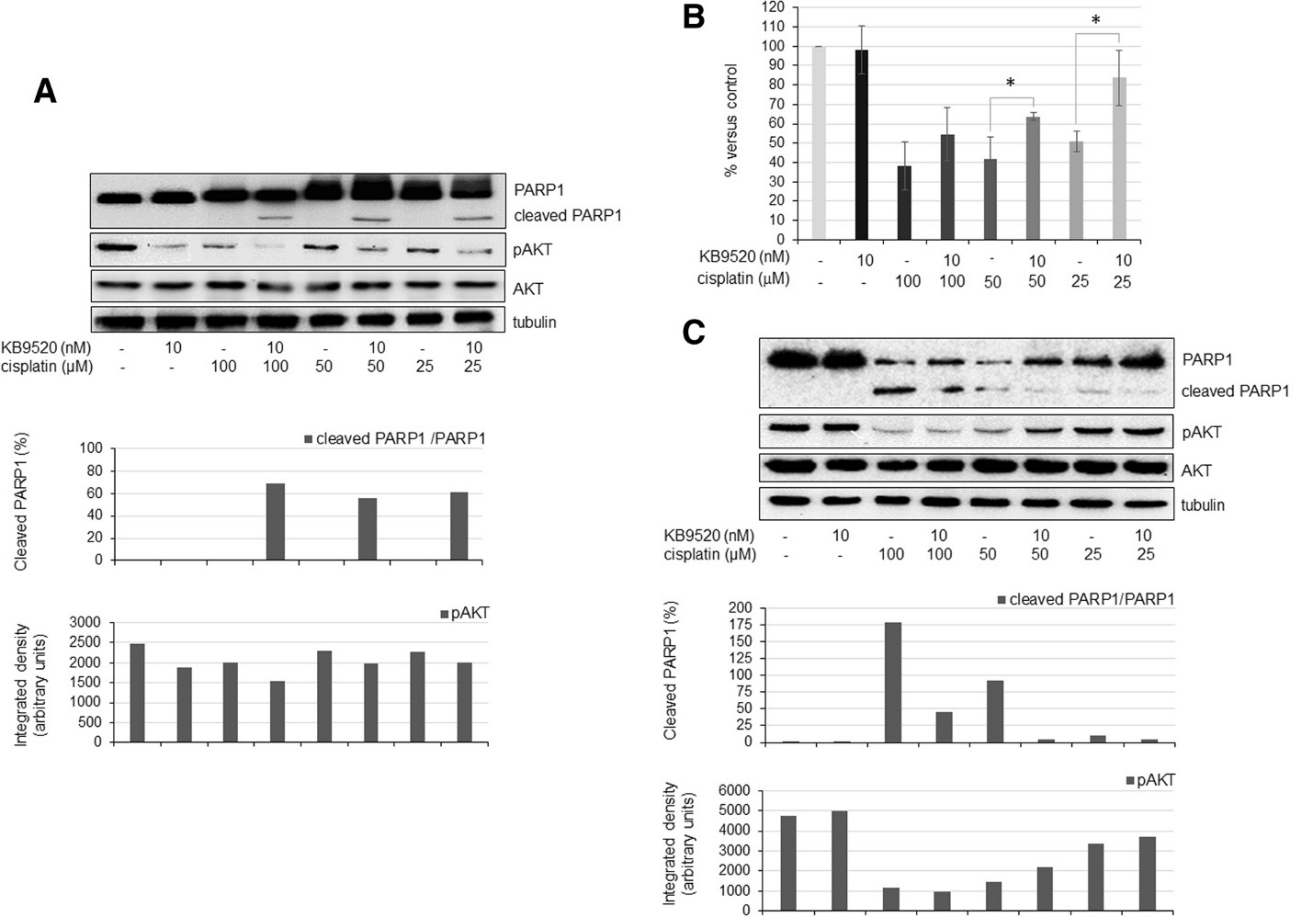
**Mechanism of KB9520 sensitization to cisplatin cytotoxicity. A)** Western blot analysis and relative densitometry of PARP1 cleavage and AKT phosphorylation in REN cells treated for 24 hours with cisplatin (25, 50 and 100 μM) or pre-treated 2 hours with KB9520 (10 nM) followed by wash-off and continued growth in normal medium ± cisplatin (25, 50 and 100 μM), for additional 24 hours. Total AKT and Tubulin staining were used for normalization. **B)** Effect on MET5A cell viability after 24 hours treatment with cisplatin (25, 50 and 100 μM) or 2 hours pre-treatment with KB9520 (10 nM) followed by wash-off and continued growth in normal medium ± different concentrations of cisplatin (25, 50 and 100 μM), for additional 24 hours. Each point represents mean ± s.d. *p ≤ 0.05. **C)** Western blot analysis and relative densitometry of PARP1 cleavage and AKT phosphorylation in MET5A cells treated with cisplatin (25, 50 and 100 μM) for 24 hours or pre-treated for 2 hours with KB9520 (10 nM) followed by wash-off and continued growth in normal medium ± different concentrations of cisplatin (25, 50 and 100 μM), for additional 24 hours. Total AKT and Tubulin staining were used for normalization. Data are representative of three separate experiments.

REN cells were treated for 24 hours with 100 μM cisplatin or pre-treated 2 hours with 10 nM KB9520 followed by wash-off and continued growth in normal medium ± 100 μM cisplatin, for additional 24 hours. After treatments, cells were stained with propidium iodide and analyzed for cellular DNA content by flow cytometry. Pre-treatment with KB9520 for 2 hours followed by 24 hours cisplatin treatment resulted in significant and efficient block in the G0/G1 phase and inhibition of cells entering the S-phase of the cell cycle compared to any other treatment (data reported in Table 2 represent mean ± s.d. (n = 3) of the percentage of cells in each phase of the cell cycle). Moreover, a significant higher percentage of dead cells were found in wells pre-treated with KB9520 followed by cisplatin compared to other treatment regimens. A plausible explanation for the higher number of dead cells in the KB9520/cisplatin treated cells was induction of apoptosis. As expected from the cell cycle analysis and percentage of dead cells, 2 hours KB9520 treatment, prior to addition of cisplatin, had the greatest effect on the appearance of cleaved PARP1 (Figure 6A). Interestingly, neither cisplatin nor KB9520 alone resulted in significant PARP1 cleavage. As increased AKT activity has been implicated in the control of proliferation, apoptosis and cisplatin resistance [44], we analyzed its activation status following different treatments. As shown in Figure 6A, KB9520 treatment significantly reduced AKT phosphorylation both in the absence and in the presence of cisplatin. The mechanism for the combined effect of KB9520 and cisplatin on AKT pathway modulation, PARP1 cleavage and increased cell death needs further studies.

**Table 2:** **Cell cycle analysis of REN cells**

Treatment/cell cycle phase	Go/G1	S	G2/M	Dead cells (sub G1)
Control	39 ± 1,9	33 ± 2,4	25 ± 0,5	3 ± 1
100 μM cisplatin (24 hrs)	39 ± 0,9	30 ± 2,3	23 ± 3,2	8 ± 2
10 nM KB9520 (2 hrs) + normal medium (24 hrs)	36 ± 1,9	29 ± 0,9	27 ± 0,9	8 ± 2
10 nM KB9520 (2 hrs) + 100 μM cisplatin (24 hrs)	48 ± 0,8	2 ± 2	28 ± 2,9	22 ± 1

### KB9520 pre-treatment protects MET5A cells to cisplatin cytotoxicity

Cisplatin is widely used in the treatment of various human solid tumors, but it is also associated with significant toxicity. We therefore tested the effect of KB9520 on cisplatin toxicity in the normal mesothelium derived cell line MET5A (Figure 6B, C). MET5A cells showed higher sensitivity to cisplatin treatment than REN cells, with an IC50 of 25 μM (Figure 6B). As previously described for REN cells, we treated MET5A cells with 10 nM KB9520 for 2 hours followed by wash-off and incubation in normal medium alone or in the presence of three concentrations of cisplatin (25, 50, 100 μM) for additional 24 hours. As reported in Figure 6B, KB9520 alone had no effect on MET5A cell proliferation or viability, while a protective effect was observed when KB9520 pre-treatment was combined with low concentrations of cisplatin. As previously described for REN cells, we analyzed PARP1 cleavage as an indicator of cell death. Western-blot analysis (Figure 6C) revealed that KB9520 pre-treatment reduced the percentage of cleaved PARP1 in cells exposed to all concentrations of cisplatin, in accordance with data obtained on cell viability. Regarding phosphorylated AKT, KB9520 had no effect on basal pAKT levels in the MET5A control cells but it antagonized the cisplatin-mediated inhibition of AKT activation (Figure 6C). AKT pathway activation is associated with anti-apoptotic effects and cell survival and together with the effects of KB9520 on reduced PARP1 cleavage, this may explain the KB9520-mediated decrease in cisplatin cytotoxicity in the non-malignant MET5A cells.

## Discussion

MPM is typically refractory to current treatment options using chemotherapy. In a first line setting, pemetrexed in combination with cisplatin has been accepted as an almost universal standard. In the second line setting, various chemotherapy agents have been used, either as monotherapy or as part of polytherapy, but none has been successfully validated.

A recently published review, based on results of a meta-analysis of unresectable MPM, suggests that response rate and survival are greater for combination therapy than for single-agent regimens, and that platinum-containing regimens have greater efficacy than non-platinum-containing combinations, confirming that platinum-based chemotherapy remains the most effective treatment for patients with MPM [45].

Our group has recently demonstrated that ERβ exerts a key role as a tumor-suppressor gene in MPM [13, 15] and its activation may also explain the gender difference clinically observed for the prognosis of MPM.

In this study we report that activating ERβ with the highly selective ligand KB9520 resulted in a concentration and time-dependent inhibition of REN malignant mesothelioma cell growth *in vitro*. Both KB9520 and E2 displayed bell-shaped responses with growth inhibition at low doses and opposite effect at high doses. Bell-shaped responses to hormones are not unusual phenomena; “hormesis” is a biphasic dose response phenomenon characterized by a low dose stressful stimulation and a high dose adaptive response (inhibition) that increases the resistance of the cell to evoked stress [46, 47]. Both ER subtypes, α and β, exert genomic and no-genomic effects which may explain the observed biphasic responses of KB9520 and E2. Other plausible explanation could be the cyclic on/off process of ER on promoters [48, 49] leading to degradation of the ERβ protein.

Although KB9520 and E2 had similar growth inhibitory effect on the REN cells there was a difference in time to maximal effect, 24 hours for KB9520 and 72 hours for E2. This time to effect difference between KB9520 and E2 may depend on the different ERβ conformational change/s induced by KB9520 and E2, respectively, which in turn may have an effect on co-regulatory protein interactions including type of co-regulatory complex, stoichiometry and kinetics.

Evaluation of the anti-proliferative effect of KB9520 on a collection of human malignant mesothelioma cell lines showed a strict dependence on ERβ expression and that the growth inhibitory efficacy was related to the level of ERβ expressed. Lack of anti-proliferative effect of KB9520 in the ERβ positive non-malignant mesothelium derived MET5A cells is difficult to understand and needs further investigation. However, the difference in the regulatory mechanisms/signaling pathways for cell growth, survival and metabolism between non-malignant and malignant cells may somehow influence the activity of ERβ and its susceptibility to respond to an ERβ agonist. Differences in post-translational modification/s of ERβ and/or cell compartment localization are plausible explanations for activity or no activity of ERβ in malignant compared to non-malignant cells. Of note though, the non-malignant MET5A cells did respond to KB9520 when stressed by cisplatin.

The enhanced growth inhibitory effect of cisplatin/pemetrexed in combination with KB9520 in REN cells *in vitro* translated to synergistic anti-tumorigenic activity *in vivo*. Treatment with KB9520 in combination with cisplatin/pemetrexed *in vivo* had greater efficacy than either treatment alone and caused a significantly reduced tumor load compared to vehicle treated animals at the end of the treatment period. Moreover, the triple combination shrunk the tumor volume even below the tumor volume at the start of treatment.

Also in a second human malignant mesothelioma cell line, MMP [13], KB9520 in complex with cisplatin resulted in synergistic growth inhibition compared to KB9520 or cisplatin alone *in vitro* (data not shown). Moreover, similar to the effect in REN cells, the combination of KB9520 and cisplatin in the MMP cells decreased the level of phosphorylated AKT and increased the levels of cleaved PARP1 (data not shown).

That a brief exposure of malignant mesothelioma cells to KB9520 (2 hours) elicited a stronger growth inhibitory effect *in vitro* compared to continuous exposure is suggestive of a hit-and-run mechanism. The long-lived biological activity of KB9520 (≥24 hours) together with a hit-and-run type of mechanism adds to our understanding of the *in vivo* tumor inhibitory efficacy of KB9520 despite its short plasma half-life of approximately 1 hour in mice.

The PI3K/AKT signaling pathway is aberrantly active and plays a critical role in cell cycle progression and cell survival in human MPM including the sensitivity to cisplatin [50]. We have previously demonstrated a role of AKT activation in MPM cell response to cisplatin [44]. Here we show that KB9520 significantly reduced pAKT levels both *in vitro* and *in vivo* (data not shown), which, at least in part, may explain the observed sensitization to cisplatin cytotoxicity. The observed reduction in AKT phosphorylation warrant further investigation; it could be due to either modulation of the rate of both protein kinase and phosphatase activities or to AKT post-translational modification that could affect its localization and/or activation status.

The order of drug administration may sometimes be very important for optimal therapeutic efficacy [51, 52]. Exposure of REN cells to KB9520 prior to cisplatin resulted in synergistic inhibition of malignant mesothelioma cell proliferation and survival whereas the reverse order of drug exposure did not even result in additive efficacy. Furthermore, KB9520 preconditioned malignant mesothelioma cells to low-concentration cisplatin cytotoxicity; combination of KB9520 with 20 μM cisplatin was as efficacious as 100 μM cisplatin alone. Thus, these data imply that KB9520, through ERβ, acts as a chemosensitizer increasing cisplatin cytotoxicity in human malignant mesothelioma cells.

Chemotherapy and in particular cisplatin is widely used in the treatment of various human solid tumors. However, cisplatin is associated with serious toxicity, which limits its use; the majority of patients diagnosed with MPM are older than 65 years and their health condition may therefore not allow the standard chemo dosing regimen of cisplatin/pemetrexed. To investigate the cytotoxic effect of the cisplatin/KB9520 combination in non-malignant mesothelial cells we treated the mesothelium-derived MET5A cells with various concentrations of cisplatin in the presence or absence of KB9520. In contrast to the effect in the malignant REN cells KB9520 diminished the toxicity of cisplatin in the MET5A cells, in part explained by the reduced PARP1 cleavage and increased pAKT levels. It has been described that ER can increase PI3K/AKT activity by interaction with the p85 subunit of PI3K [53, 54]. However, if that explains the increased pAKT levels in the MET5A cells needs to be explored in more detail.

## Conclusions

In summary, in this report we have shown that MPM cell proliferation and tumor growth can be effectively suppressed by selective agonist activation of ERβ. We have also shown that KB9520 acts as a chemosensitizer through activation of ERβ and that the order of drug administration in combination with cisplatin/pemetrexed is essential for the synergistic efficacy observed *in vitro* and *in vivo*. KB9520 had no cytotoxic effect in the ERβ expressing non-malignant mesothelium derived MET5A cells. In contrast, it diminished cisplatin cytotoxicity in these cells. Thus, combination of KB9520 with SOC (cisplatin/pemetrexed combination) may increase the sensitivity of MPM tumors to the SOC regimen in patients and perhaps result in higher response rates, extended progression free survival (PFS) and prolonged overall survival (OS), without adding toxicity. Furthermore, combination with KB9520 may allow milder SOC (cisplatin) regimen without loss of anti-tumor efficacy and thereby may become an option for patients that cannot tolerate the standard and more aggressive cisplatin/pemetrexed dose regimen.

## Methods

### Reagents and antibodies

The monoclonal antibodies specific for α-Tubulin, PARP1 and the polyclonal antibody specific for ERβ were purchased from Santa Cruz Biotechnology (Santa Cruz, CA, USA). Phospho-AKT (pSer473) was from Cell Signaling Technology (Beverly, MA, USA), anti-mouse and anti-rabbit IgG peroxidase conjugated antibodies and chemical reagents were from Sigma-Aldrich (St Louis, MO, USA). ECL was from Amersham Pharmacia Biotech (Uppsala, Sweden). Nitrocellulose membranes and protein assay kit were from Bio-Rad (Hercules, CA, USA). Culture media, sera, antibiotics and LipofectAMINE transfection reagent were from Invitrogen (Carlsbad, CA, USA). The ERβ selective agonist KB9520 (see Additional file 1: Figure S1) was designed and synthesized by Karo Bio (Huddinge, Sweden). (KB9520 has been described previously [36, 41–43]. The compound can be obtained following contact with Karo Bio AB [stefan.nilsson@karobio.se] and after signing of a Material Transfer Agreement together with a detailed protocol of planned study. A fee covering the cost of compound synthesis will be charged).

### Cell cultures and transfection

The epithelioid MPM derived REN cell line, used as the principal experimental model in this investigation, was isolated, characterized and kindly provided by Dr. Albelda S.M. (University of Pennsylvania, Philadelphia; PA, USA). Cells were characterized by BMR Genomics s.r.l. using the PowerPlex 18D System kit. The biphasic MSTO-211H and the mesothelial MET5A cell lines were obtained from the Istituto Scientifico Tumori (IST) Cell-bank, Genoa, Italy; the MMB cell line derived from pleural effusions of patients with MPM and stabilized in culture [55]; the H2596 cell line produced by Dr. H. I. Pass from surgical specimens derived from patients with resected MPM [56] were kindly provided by Dr. W. Thomas (RCSI, Dublin, IRL) in 2011. Cells were grown in RPMI medium supplemented with 10% FBS, 100 μg/ml streptomycin and 10 μg/ml penicillin at 37°C in a humidified environment containing 5% CO2. Mycoplasma infection was excluded by the use of Mycoplasma PlusTM PCR Primer Set kit from Stratagene (La Jolla, CA, USA). Cells grown to 80% confluence in tissue culture dishes were transiently transfected with the pCNX2 plasmid expressing human wild type ERβ (Addgene, Cambridge, MA, USA) using LipofectAMINE reagent as described by the manufacturer.

Gene silencing was achieved using an ERβ-specific shRNA lentiviral plasmid (pLKO.1-puro) by Sigma (St Louis, MO, USA) or specific siRNAs by Qiagen (Hilden, Germany).

### Proliferation assays

Cells were seeded at a density of 10×10^4^ cells/well in 6-well plates in RPMI medium supplemented with 10% FBS, 100 μg/ml streptomycin and 10 μg/ml penicillin and incubated over-night at 37°C in a humidified environment containing 5% CO2 to allow adherence. Following treatment cells were trypsinized and stained with Trypan blue. The number of cells considered viable (unstained cells) was counted in a Bürker haemocytometer within 5 minutes after staining.

### Wash-off experiments

Cell cultures were pretreated with KB9520 for 1–16 hours (depending on experiment) followed by wash-off and then replenished with normal growth medium ± cisplatin, pemetrexed or cisplatin/pemetrexed. Total incubation time was 24–72 hours (depending on experiment). Control cultures were maintained in normal growth medium without added drug. The number of viable cells was determined as described. For details of each experiment see figure legends.

### Add-on experiments

In the add-on experiments the second drug was added directly to the cell culture medium without wash-off of the first drug. Total incubation time was 24 hours. Control cultures were maintained in normal growth medium without added drug. The number of viable cells was determined as described.

### Cell lysis and immunoblot

Cells were extracted with 1% NP-40 lysis buffer (1% NP-40, 150 mM NaCl, 50 mM Tris–HCl pH 8.5 mM EDTA, 10 mM NaF, 10 mM Na_4_P_2_O_7_, 0.4 mM Na_3_VO_4_) with freshly added protease inhibitors (10 μg/ml leupeptin, 4 μg/ml pepstatin and 0.1 Unit/ml aprotinin). Lysates were centrifuged at 13.000 × g for 10 minutes at 4°C and the supernatants were collected and assayed for protein concentration with the Bio-Rad protein assay method.

Proteins were separated by SDS-PAGE under reducing conditions. Following SDS-PAGE, proteins were transferred to nitrocellulose, reacted with specific antibodies and then detected with peroxidase-conjugate secondary antibodies and chemiluminescent ECL reagent. Densitometric analysis was performed using the GS 250 Molecular Image (Bio-Rad).

### Cell cycle analysis

For cell cycle/apoptosis analysis, 5 × 10^5^ cells were seeded in tissue culture plates and treated with 10 nM KB9520, 100 μM cisplatin or the combination of the two drugs for 24 hours at 37°C in a 5% CO2 atmosphere. After incubation, detached and suspended cells were harvested in complete RPMI and centrifuged at 500 × g for 10 minutes. Pellets were washed with PBS, fixed in ice-cold 75% ethanol at 4°C, treated with 100 mg/mL RNAse A for 1 hour at 37°C, stained with 25 μg/mL propidium iodide and finally analyzed by using a flow cytometer FACS (Becton Dickinson, San Jose, CA, USA) and Modfit software (Verity Software House, Topsham, ME, USA).

### In vivo experiments

#### Animals

CD1 nude mice (males, 6 weeks old; Charles River, Calco, Italy) received intra-peritoneal (i.p.) injections of 2×10^6^ luciferase transduced REN cells in 0.5 mL of RPMI medium. After anesthetization and i.p. injections of 0.3 mL of 15 mg/mL D-luciferin, tumor dimension and localization of luminescent cells was monitored using the In Vivo Imaging System (IVIS®) system 100 series (Xenogen Corporation, Hopkinton, MA, USA). Regions of interest were identified around the tumor sites and were quantified as total photon counts using Living Image software (Xenogen Corporation). The values of tumor sizes were obtained by subtracting luminescence signals of each weekly measurement by the average of all animals within a treatment group on the 15^th^ day after inoculation (day when treatment started). To evaluate treatment toxicity, mice were weighed at the start and end of treatments. Mice were killed and necropsied after 20 days of treatment. *In vivo* experiments were approved by Istituto Scientifico Tumori (Genoa, Italy) ethical committee and conform to the relevant regulatory standards. Mice were maintained and handled under aseptic conditions, and were allowed access to food and water ad libitum.

#### Drug administration

An elapse of 15 days was allowed for the formation of detectable tumor nodules, assessed by IVIS® imaging. Mice were then weighed and stratified into treatment groups of ten animals. Treatment protocols were done from the 15^th^ day to the 35^th^ day, and mice were analyzed weekly by IVIS® imaging to assess tumor growth. One dose of KB9520 was used (10 mg/kg/day). KB9520 was dissolved in the vehicle (5% DMSO/40% PEG 400/55% water) and administrated once daily (days 15–35) by sub-cutaneous administration. 5 mg/kg cisplatin solution (Ebewe Italia srl, Roma, Italy) was administrated subcutaneously at day 18 and 25, respectively, and 150 mg/kg pemetrexed (dissolved in isotonic saline) (Eli Lilly, Houten, Nederland) was injected subcutaneously at days 19–23 and 26–30, respectively. Untreated animals were dosed with empty vehicle. At day 35 mice from the four groups were euthanized and necropsied. Tumors growing in the peritoneum were excised, and one part of the tumor tissues was immediately frozen and stored at −80°C for subsequent analysis.

### Statistical analysis

Statistical evaluation of the differential analysis was performed by one way ANOVA and Student’s t-test. The threshold for statistical significance was set at p ≤ 0.05. The statistical analysis of *in vivo* experiments was done by using R [57]. To compare different groups we used the non-parametric Kruskal-Wallis test; if differences were found significant (p ≤ 0.05) we subsequently applied the Wilkoxon rank sum test to do the pair wise comparisons. To compare tumor growth curves we used the StatMod package.

## Electronic supplementary material

Additional file 1: Figure S1: Concentration-dependent induction of reporter gene expression in 293 cells genetically engineered to express the human estrogen receptor alpha (hERα) and human estrogen receptor beta 1 (hERβ) [58]. (PDF 119 KB)
