# Supplementary material for: Agonist activation of estrogen receptor beta (ERβ) sensitizes malignant pleural mesothelioma cells to cisplatin cytotoxicity
Source: Mol Cancer. 2014 Oct 2;13:227. doi: 10.1186/1476-4598-13-227 (PMC4197308; doi:10.1186/1476-4598-13-227)
Supplement: Supplementary file 1 — Additional file 1: Figure S1: Concentration-dependent induction of reporter gene expression in 293 cells genetically engineered to express the human estrogen receptor alpha (hERα) and human estrogen receptor beta 1 (hERβ) [58]. (PDF 119 KB) [file 12943_2014_1428_MOESM1_ESM.pdf]

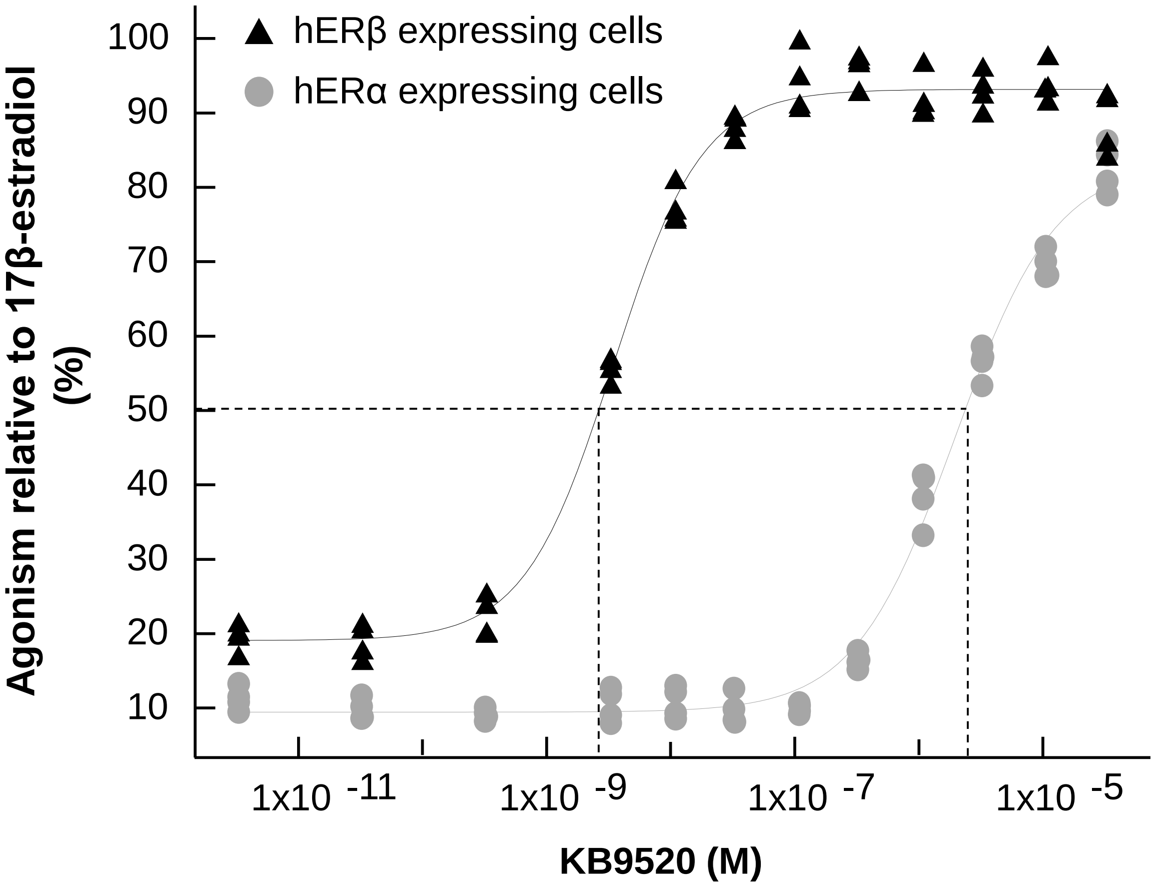

Figure S1. Concentration-dependent induction of reporter gene expression in 293 cells genetically engineered to express the human estrogen receptor alpha (hERα) and human estrogen receptor beta 1 (hERβ) [58].
